# A Novel Eco-Friendly Wood Adhesive Composed by Sucrose and Ammonium Dihydrogen Phosphate

**DOI:** 10.3390/polym10111251

**Published:** 2018-11-12

**Authors:** Zhongyuan Zhao, Shin Hayashi, Wei Xu, Zhihui Wu, Soichi Tanaka, Shijing Sun, Min Zhang, Kozo Kanayama, Kenji Umemura

**Affiliations:** 1 Co-Innovation Center of Efficient Processing and Utilization of Forest Resources, Nanjing Forestry University, Nanjing 210037, China; 2 College of Furnishings and Industrial Design, Nanjing Forestry University, Nanjing 210037, China; xuwei@njfu.edu.cn (W.X.); Wzh550@sina.com (Z.W.); 3 Laboratory of Sustainable Materials, Research Institute for Sustainable Humanosphere, Kyoto University, Gokasho, Uji, Kyoto 611-0011, Japan; hayashi.shin001@jp.panasonic.com (S.H.); soichi_tanaka@rish.kyoto-u.ac.jp (S.T.); zhang888@rish.kyoto-u.ac.jp (M.Z.); kozo-kanayama@rish.kyoto-u.ac.jp (K.K.); 4 College of Material Science and Engineering, Nanjing Forestry University, Nanjing 210037, China; Sunsj-611@163.com

**Keywords:** eco-friendly adhesive, sucrose, particleboard

## Abstract

Development of a bio-based wood adhesive is a significant goal for several wood-based material industries. In this study, a novel adhesive based upon sucrose and ammonium dihydrogen phosphate (ADP) was formulated in hopes of furthering this industrial goal through realization of a sustainable adhesive with mechanical properties and water resistance comparable to the synthetic resins used today. Finished particleboards exhibited excellent mechanical properties and water resistance at the revealed optimal adhesive conditions. In fact, the board properties fulfilled in principle the requirements of JIS A 5908 18 type standard, however this occured at production conditions for the actual state of development as reported here, which are still different to usual industrial conditions. Thermal analysis revealed addition of ADP resulted in decreases to the thermal thresholds associated with degradation and curing of sucrose. Spectral results of FT-IR elucidated that furanic ring chemistry was involved during adhesive curing. A possible polycondensation reaction pathway was proposed from this data in an attempt to explain why the adhesive exhibited such favorable bonding properties.

## 1. Introduction

Biomaterial-based construction products such as particleboard, plywood, fiberboard and other composites are typically used in our living environments [1]. Most of these materials are manufactured by bonding the wooden elements together using synthetic resin adhesives. Examples of such resin adhesives include formaldehyde-based, isocyanate-based, vinyl acetate-based resins and so on, in which, many wood-based materials bonded by formaldehyde-based adhesives [2,3,4]. Recently, regulatory and consumer concerns about both formaldehyde emissions and a present dependency upon fossil-derived resources has spurred the wood industry to develop eco-friendly adhesives formulated from renewable and bio-resources based materials. Examples of eco-friendly wood adhesives include those that are lignin-based, soybean-based, starch-based, tannin-based, and more [5,6,7,8]. In research of the eco-friendly wood adhesives, the first efforts were to partially-substitute synthetic resin adhesives by the renewable substances to make the wood adhesives more sustainable, but it was not completely sustainable. Therefore, since this method did not address the issues of sustainability, focus has shifted towards preparing resins entirely comprised of sustainable materials, which attained the bond performance equal to or greater than existing synthetic resins [9,10].

Recently, some previous research indicated that the sucrose could be used as a raw material for wood adhesives. The disaccharide sucrose is in fact one of the most abundantly available renewable chemicals in the world, which primarily produced from beet and sugarcane, and it is available at a very high level of purity and at low cost [11,12,13]. The principal utilization of sucrose is as a sweetener for the food and beverage industry. With the recent scientific advent of establishing “green chemistry”, sucrose has continuously been identified as a promising alternative to non-renewable resources, and the applications for sucrose can be found in various chemical and processing industries, such as fine chemicals [11], bioethanol [12], surfactants [13] and many more. Because of this, the trend regarding the utilization of sucrose, existing and active research about sucrose chemistry focuses upon the preparation and utilization of products derived from sucrose. 5-hydroxymethyl-2-furfural (5-HMF) is considered as one of the most important sucrose-derived products, which is a significant platform chemical for production of a variety of plastics and polymers. Due to its di-functionality (hydroxyl and aldehyde groups), 5-HMF is convertible into a variety of chemicals through means such as functionalization via oxidation, reduction, hydrogenolysis, and condensation. Some examples of useful chemical derivatives of 5-HMF include levulinic acid (LA), 5-ethoxymethylfurfural (EMF), 2,5-furandicarboxaldehyde (DFF), 2,5-furandimethanol (FDM), 5-formyl-2-furancarboxylic acid (FFCA), furan-2,5-dicarboxylic acid (FDCA), 5-Hydroxymethyl-2-furancarboxylic acid (HMFCA), maleic anhydride (MA), and so on [14,15,16,17,18].

Based on the reactive chemical properties of 5-HMF, we have focused upon incorporating 5-HMF derived from sucrose into various adhesive systems, e.g., citric acid-sucrose, tannin-sucrose, tannin-sucrose-citric acid, and tannin-sucrose-sulfuric acid adhesives [19,20,21,22]. The intended application for these adhesives is as an eco-friendly wood adhesive for particleboard manufacturing. However, these adhesives were found to require very low pH values for production, which was due to acidic catalysis of sucrose to form 5-HMF without significant addition of temperature. Problematically, this low pH requirements may pose corrosion risks of the metal which used on the manufacturing process of wood-based materials. To address these acid-derived considerations, we shifted focus to application of phosphate catalysts for influencing sucrose conversion reactions. Recently, we found that sucrose and ammonium dihydrogen phosphate (ADP) could form an insoluble polymer [23]. However, the details concerning manufacturing conditions, final bonding properties in wood-based materials, and the curing mechanism were not investigated. To follow up on this topic, the present study addresses several fine details regarding sucrose-ADP adhesive formulation for application as a binder for wood-based products.

## 2. Materials and Methods

### 2.1. Materials

Sucrose (guaranteed reagent) and ammonium dihydrogen phosphate (ADP, guaranteed reagent) were purchased from Nakalai Tesque, Inc. (Kyoto, Japan), and used as received without further purification. The reagents were vacuum-dried at 60 °C for 15 h until constant mass prior to usage in experiments. Recycled wood particles were obtained from a particleboard company in Japan. The wood particles were screened by a sieving machine to collect particles between the aperture sizes of 5.9 and 0.9 mm. Before particleboard manufacturing, particles (original moisture content, 3 to 4 wt %) were dried in an oven at 80 °C for 12 h to reach a final moisture content of 2 wt %.

### 2.2. Preparation of Sucrose-ADP (SADP) Adhesives

Sucrose and ADP were dissolved in distilled water at various proportions, and the concentration of the solution was adjusted to 50 wt %. The viscosity and pH of the solution were measured by Viscolead One (Fungilab, Barcelona, Spain) at 20 °C and Horiba pH meter D-51, respectively, and the results are shown in Table 1. It’s worth noting that the viscosity of SADP adhesives are very low, that is due to the particleboard manufacture method, in which, lower viscosity is benefit for spraying adhesive on the particles. However, the low viscosity can possibly lead to the absorption by wood particles, which might have influence on the particleboard properties. This topic should be clarified in further research.

### 2.3. Manufacture of Particleboards

The adhesive solutions were sprayed onto wood particles in a blender, at the resin content (solid content) at 20 wt % (calculated based on the weight of oven dried wood particles). The sprayed particles were then dried at 80 °C for 12 h until the moisture content was 4 to 7 wt % (In the explore research, the particleboards were manufactured without drying treatment, however, the mechanical properties were lower than the requirements, therefore a second drying treatment was utilized in this research). Dried particles were mat-formed using a forming box of 300 × 300 mm. The particleboards were manufactured under following two conditions: (a) The mats mixed by various adhesives were hot-pressed at 180 °C for 10 min; (b) The mats were mixed with SADP 85/15 ratio adhesive only, and hot pressed at 140, 160, 180, 200 and 220 °C for 10 min. The basic manufacture information of two types particleboards is shown in Table 2. The size of all the manufactured board was 300 × 300 × 9 mm, and the target density was 800 kg/m^3^.

### 2.4. Evaluation of Particleboard Properties

Particleboards were conditioned for 1 week at 20 °C and 60% relative humidity (RH), and then evaluated according to the Japanese Industrial Standard for particleboard (JIS A 5908, 2003) [24]. The static 3-point bending test was carried out on a 200 mm × 30 mm × 9 mm specimen from each board, and the effective span and loading speed were 150 mm and 10 mm/min, respectively. The modulus of rupture (MOR) and the modulus of elasticity (MOE) were calculated from the bending test. The internal bond strength (IB) test was performed on a 50 mm × 50 mm specimen with a loading speed of 2 mm/min, and thickness swelling (TS) after water immersion for 24 h at 20 °C was measured in specimens of the same size. In the TS test, each sample was immersed in 200 ml water, and pH values of the soaked water were measured. After the TS test, the specimens were subjected to a cyclic aging treatment (drying at 105 °C for 10 h, warm-water immersion at 70 °C for 24 h, drying at 105 °C for 10 h, immersion in boiling water for 4 h, and drying at 105 °C for 10 h). The thickness and the weight changes of the specimens that occurred throughout the treatments were determined. Each experiment was performed five times, and the average values and standard deviations were calculated. Statistical significance was considered for *p* values < 0.05.

### 2.5. Thermal Analysis

To prepare powdered adhesive for the further research, 20 g of SADP 85/15 adhesive, sucrose only, and ADP only solutions with 50 wt % concentration, were divided into 10 aluminum caps and dried in an oven at 80 °C for 12 h to obtain the uncured adhesive for next step. The samples were analyzed by thermogravimetric analysis (TGA) and differential scanning calorimetry (DSC) using a TGA 2050 (TA Instruments, Tokyo, Japan) and DSC 2910 (TA Instruments, Tokyo, Japan), respectively. The powders were scanned from room temperature to 400 °C at a rate of 10 °C/min under nitrogen purging.

### 2.6. Measurement of Insoluble Mass Proportion

The SADP 85/15 and sucrose powders were divided into 5 parts and heated at 120, 140, 160, 180, and 200 °C for 10 min to prepare heated samples. 2 g of each heated sample were then boiled in distilled water for 4 h to obtain insoluble mass, the results were obtained by the average of triplicated boiling on three different repetitions of the same sample. The obtained insoluble mass was vacuum-dried at 60 °C until constant mass (~15 h) and finally weighed. The insoluble mass proportion (IMP) was calculated by the following equation:

(1) $$\mathrm{IMP}\;\left(\%\right)=\frac{Weight\;of\;dried\;insoluble\;mass\;\left(g\right)}{Weight\;of\;heated\;sample\left(g\right)}\times 100\%$$

### 2.7. Fourier Transform Infrared Spectra

Fourier transform infrared spectra were acquired to probe for chemical changes to the insoluble mass of SADP 85/15 adhesive cured at different heating temperatures for 10 min. The spectra of SADP uncured adhesive was obtained as a control. Infrared spectra were obtained using a Fourier transform infrared spectrophotometer (FT/IR-4200, JASCO Corporation, Tokyo, Japan) by the KBr disk method, and recorded with an average of 32 scans at a resolution of 4 cm^−1^.

## 3. Results and Discussion

### 3.1. Effects of Mixture Proportions on Particleboard Properties

The SADP adhesives with different mixture proportions were utilized to manufacture the particleboard and the bonding properties were investigated. First, particleboards were manufactured using identical hot-pressing conditions (180 °C, 10 min, 20 wt % resin content) to gauge the effects of ADP proportions on the bonding properties.

**Figure 1:**
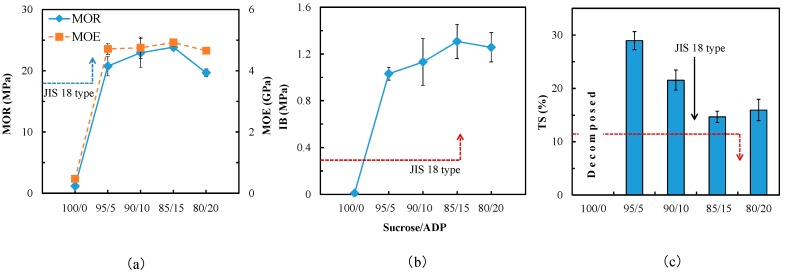
Properties of the particleboards bonded with SADP adhesives at different proportions. (**a**) Bending properties; (**b**) Internal bonding strength; (**c**) Thickness swelling. Error bars indicate the standard deviations.

Particleboard mechanical properties and thickness swelling are shown in Figure 1. As a control, the board formulated with pure sucrose exhibited MOR of 1.17 MPa but no measurable IB, and the board destroyed after the TS test. This indicates that essentially zero benefit is gained from an adhesive based solely upon sucrose. Beginning with 5 wt % addition of ADP, the mechanical properties measured demonstrated excellent bending performance (MOR 20.76 MPa, MOE 4.7 GPa, IB 1.03 MPa). The MOR, MOE and IB average values exhibited a rising trend as the ADP content was increased from 5–15 wt %, although variance analysis (ANOVA) revealed no significant (*p* > 0.05) difference of MOE between the particleboards manufactured with SADP 95/5 and 90/10. However, a slightly decrease in board properties was observed from the SADP 80/20 final product. There are possible two reasons caused this observation. First, sucrose was considered to contribute the main bonding strength in this adhesion system, therefore, as the reducing of sucrose content, the physical properties of the particleboard decreased. Secondly, when the proportion between sucrose and ADP at 85/15, the polymerization reaction was sufficient, therefore, when the proportion was 80/20, the added ADP did not participate in the reaction, which led to the physical properties decreasing.

Nevertheless, the mechanical properties of all the samples bonded by SADP systems satisfied the requirement of JIS A5908 type 18 standard. In the results of TS (Figure 1c), a trend similar to what was observed for mechanical properties could be found. The optimal TS value (14.6%) was measured in the board bonded with SADP 85/15, which was still greater than the amount allowed by JIS 18 type standard (<12%), and this indicates that the water resistance of SADP adhesive need to be improved. To verify the acidity of particleboards, the pH values of the soaking water from the TS tests was determined. As shown in Table 3, increases to ADP content resulted in decreasing pH values in the soaking water. This means that the acidity of the particleboard was increasing due to the addition of ADP. Therefore, higher dosages of ADP resulted in greater extents of phosphoric acid genesis during hot pressing. This also could be considered as evidence for explaining the deteriorating mechanical properties of the boards bonded by SADP 80/20.

**Figure 2:**
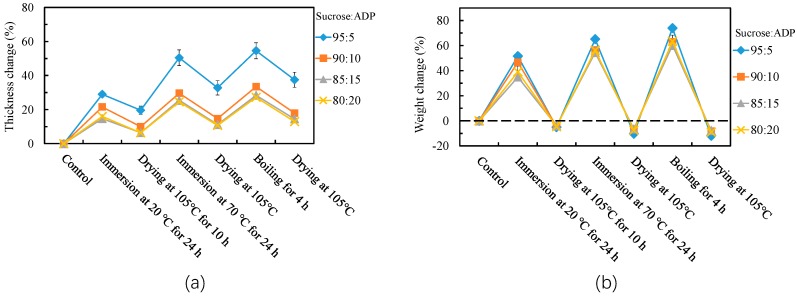
Thickness change (**a**) and weight change (**b**) during cyclic aging treatment of the particleboards bonded with SADP adhesives with different proportions. Error bars indicate the standard deviations.

The results of thickness and weight changes of cyclic aging treatments are shown in Figure 2a,b, respectively. Regarding thickness changes, the extent of thickness change at each stage of the cyclic aging treatment decreased as the increasing of ADP content. ANOVA analysis showed no significant difference in the thickness changes of all treatments between the boards bonded by SADP 85/15 and SADP 80/20. The lowest thickness changes of the second (Immersion at 70 °C for 24 h) and third (Boiling for 4 h) immersion treatments were 25% and 27%, respectively, which were lower than values (29% and 32%) obtained from the particleboard bonded by pMDI adhesive (Manufacture conditions: Thickness of board was 9 mm; Resin content was 8 wt %; Hot pressing temperature was 200 °C; Hot pressing time was 10 min; Density was 800 kg/m^3^) [20], indicating that the boards bonded with SADP adhesives exhibited strong dimensional stability. Figure 2b shows that when the ADP content of the adhesive at 5% to 15%, the weight change fluctuation of the boards decreased by increasing ADP content, indicating that the addition of ADP improved the inhibition of water absorption in this content range, the lowest range was derived from the board bonded by SADP 85/15 (−8% to 60%). However, a slight increase of the weight change fluctuation were observed from the board bonded by SADP 80/20 (−9% to 63%), which was possible due to the dissolution of superfluous ADP. Judging from the results of the effects of mixture proportion on the particleboard properties, it can be stated that the most effected SADP adhesive was prepared at the proportions of 85/15. Therefore, this adhesive recipe was utilized henceforth throughout this report.

### 3.2. Effects of Hot Pressing Temperature on Particleboard Properties

**Figure 3:**
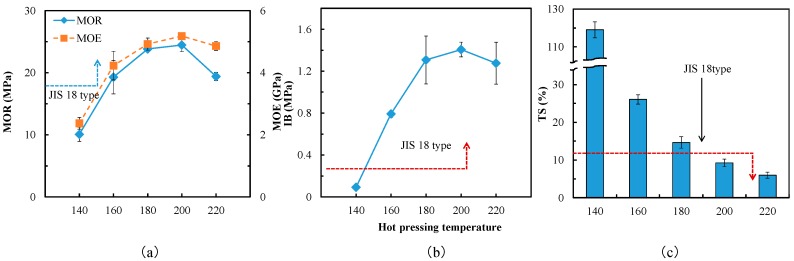
Properties of the particleboards bonded with SADP 85/15 adhesive at different hot pressing temperatures. (**a**) Bending properties; (**b**) Internal bonding strength; (**c**) Thickness swelling. Error bars indicate the standard deviations.

The effects of hot pressing temperature on the properties of particleboard bonded with SADP 85/15 adhesive is shown in Figure 3. When hot press temperature was in the range of 140–200 °C, the boards exhibited a positive correlation between mechanical properties and hot pressing temperatures. The maximum property values were obtained at 200 °C (MOR = 24.46 MPa, MOE = 5.2 GPa, IB = 1.4 MPa). A slight decrease to mechanical properties was observed at 220 °C, this was possibly caused by a pronounced effect of ADP pyrolysis during board production. This is because the phosphoric acid and pyrophosphoric acid produced during ADP pyrolysis (Equations (1) and (2)) [25] may have induced some decomposition of the structural polysaccharides within the finished particleboard [26].

(2) $$\;({\mathrm{NH}}_{4})H_{2}{\mathrm{PO}}_{4}\overset{∆}{\to }{\mathrm{NH}}_{3}↑+H_{3}{\mathrm{PO}}_{4}\;$$

(3) $$\;2H_{3}{\mathrm{PO}}_{4}\overset{∆}{\to }H_{2}O+H_{4}P_{2}O_{7}\;$$

when the hot-pressing temperature was equal to or greater than 160 °C, the mechanical properties of the particleboards fulfilled the requirement of JIS A5908 type standard. For the results of the TS experiments, a positive correlation between water resistance and hot pressing temperature was obtained. The lowest TS value was observed for the board bonded at 220 °C (TS 5.9%), which exhibited excellent dimensional stability characteristics. Considering the reason of mechanical properties decrease, the reducing of TS also could be attributed to the decomposition of the structural polysaccharides (such as cellulose or hemicellulose), which reduced the hydroxide of particles. The TS of the board formulated at 200 °C was 9.2%, which also satisfied the JIS A 5908 standard.

**Figure 4:**
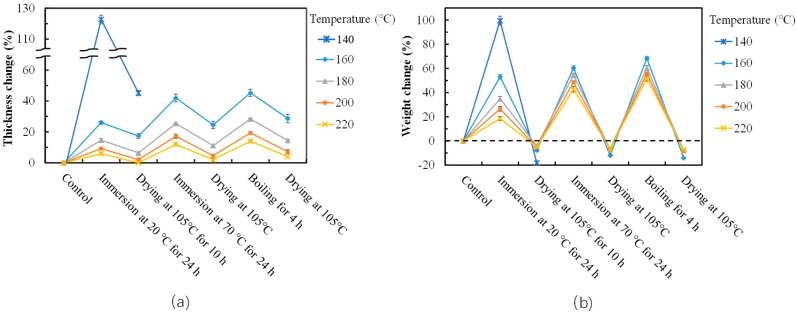
Thickness change (**a**) and weight change (**b**) during cyclic aging treatment of the particleboards bonded with SADP 85/15 adhesives with different hot pressing temperatures. Error bars indicate the standard deviations.

Thickness and weight changes in the cyclic aging treatments are shown in Figure 4. Most of the samples could maintain their shape after the treatments. One exception was observed in the particleboard bonded at 140 °C. A logical explanation for this exception is that this temperature of hot pressing lead to insufficient adhesive curing. The thickness and weight change region of the particleboards decreased with increasing hot pressing temperatures, indicating that temperature is a significant factor for water resistance imparted by the applied SADP 85/15 adhesive. Based on the evaluation results of the particleboard bonded at various conditions, the optimal hot pressing temperature for the tested SADP adhesive was concluded to be 200 °C.

### 3.3. Thermal Analysis

#### 3.3.1. TG Analysis

**Figure 5:**
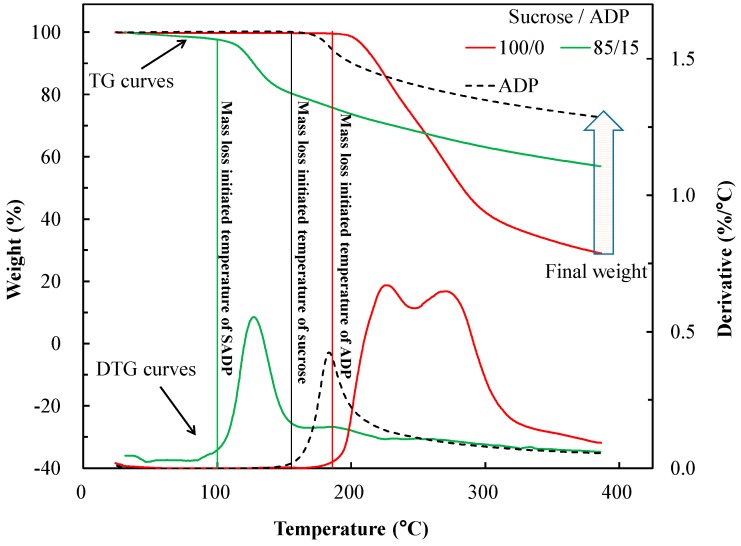
TG and DTG curves of sucrose (100/0), ADP (0/100), and uncured SAPD adhesive (85/15).

To investigate the curing behavior during the heating treatment, the thermal analysis and measurement of insoluble mass proportion were carried out. Figure 5 shows the thermogravimetric (TG) and derivative TG (DTG) curves of the SADP 85/15 adhesive. To serve as a control, 100% sucrose and 100% ADP were also analyzed. From the TG results, it can be seen that sucrose exhibited rapid mass loss starting at 195 °C. This was due to heat-induced caramelization reactions [27]. For ADP, degradation was initiated at around 150 °C. This thermal event could be attributed to its pyrolysis [28,29]. However, when analyzing the 85/15 adhesive, the preliminary weight loss moved to lower temperatures (around 100 °C). It is possible that this was due to catalysis by ADP towards thermal degradation or the caramelization of sucrose. In addition, when the temperature reached approximately 260 °C, the mass loss of SADP 85/15 was higher than for pure sucrose (100/0), indicating that the addition of ADP decreased the starting temperature for the mass loss. Besides that, the final residual mass after the TG analysis of sucrose was 29%, while the values of ADP and SADP 85/15 were 73% and 57%, respectively. These observations indicate that the addition of ADP improved the thermal stability of sucrose at high temperature.

From the DTG curves, it was observed that pure sucrose underwent two-step degradation. The first instance of degradation was observed at 220 °C, previously defined as being related to the caramelization of sucrose [30]. The second step of degradation occurred at around 270 °C, this have been attributed to formation of a black, aerated, and char-like solid [27]. For ADP, a one-step thermal degradation was observed at 185 °C, which was considered to be ammonia-producing decomposition [28]. Moving on to the SADP 85/15, it was observed that the addition of ADP resulted in decreasing temperature of weight loss, and one-step degradation was observed at 128 °C, which was significantly lower than those of sucrose and ADP only.

#### 3.3.2. DSC Analysis

**Figure 6:**
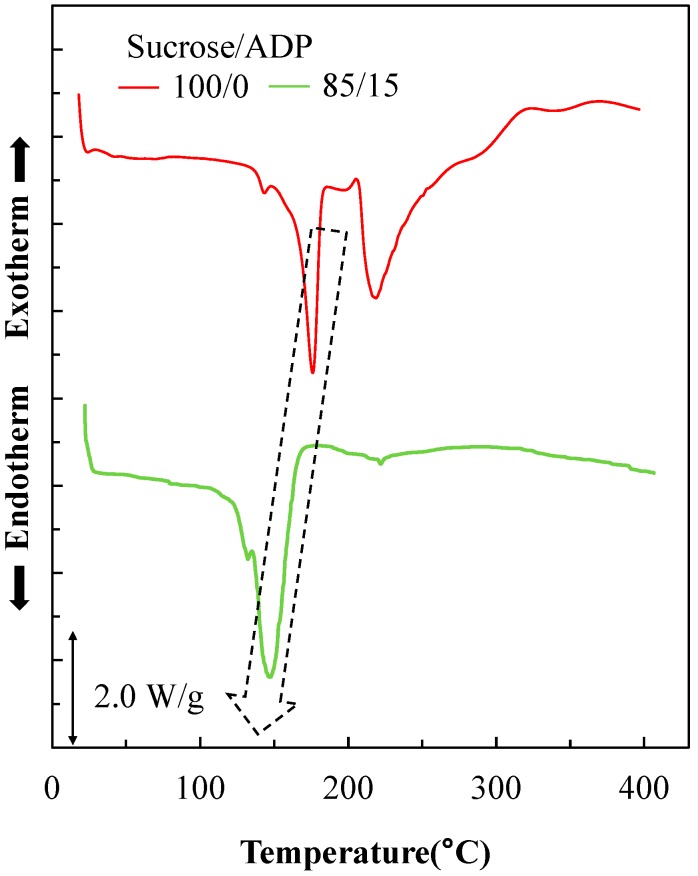
DSC curves of sucrose and SADP 85/15 adhesive.

Figure 6 displays DSC curves obtained from pure sucrose and the SADP 85/15 adhesive. First, sucrose alone showed two endothermic peaks located at 180 and 220 °C. Based on the fact that TG analysis did not show any mass loss at 180 °C, those peaks must be due to melting and caramelization of sucrose, respectively [27,31]. For the SADP 85/15 adhesives, it was observed that the addition of ADP resulted in endotherm peaks located at lower temperatures (146 °C). Since the TG analysis showed a mass loss at that temperature, this endothermic reaction is linked with the decomposition of sucrose under the catalysis of ADP. Furthermore, the peaks derived from sucrose were hardly observed over 200 °C, indicating that some endothermic reactions occurred between sucrose and ADP at this temperature.

Based on the results of TG and DSC analysis, the degradation and endothermic reaction temperature of sucrose reduced by adding ADP. Furthermore, judging from the results of the thermal analysis, the characteristic peaks of sucrose and ADP could not be observed, indicating chemical reactions taking place which involve interactions between sucrose and ADP.

### 3.4. Insoluble Mass Proportion

**Figure 7:**
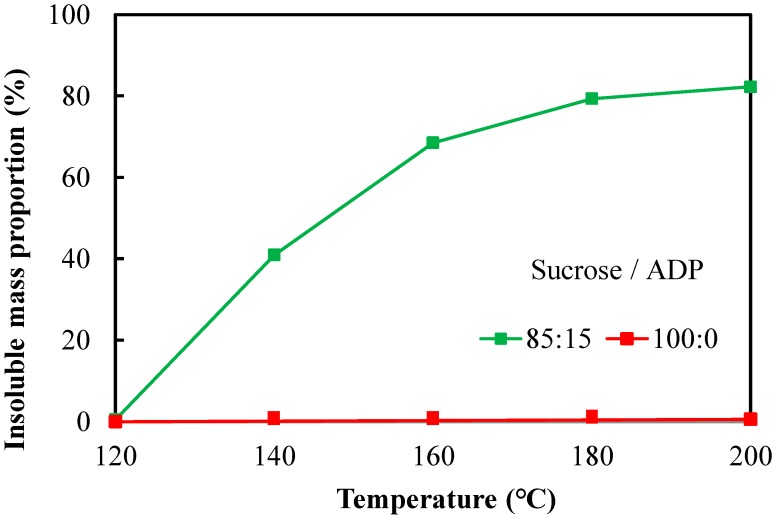
Insoluble mass proportion of SADP 85/15 adhesive and sucrose under different temperatures.

To investigate the relationship between the thermal reaction and curing behavior of SADP 85/15 adhesive, the measurement of insoluble mass proportion was carried out. The results are shown in Figure 7, and the standard deviation is in the range of 0.6% to 1.3%. The insoluble mass proportion of sucrose (100/0) was hardly observed, irrespective of heating temperatures, indicating that when sucrose is heated below 200 °C, all of its products remain water- soluble. Across SADP 85/15 samples, increasing heating temperature resulted in an increase to insoluble mass proportions, indicating a positive correlation between temperature and insoluble mass proportion, and the highest value obtained at 200 °C (82%). When the heating temperature was 120 °C, zero insoluble mass proportion was obtained, which was possible due to the insufficient curing of the adhesive. Comparing with sucrose only condition, some insoluble mass was obtained at lower temperatures through addition of ADP. Based on the results of evaluation of board properties, these insoluble mass contributed to the water resistance of the particleboards.

### 3.5. FT-IR Analysis

**Figure 8:**
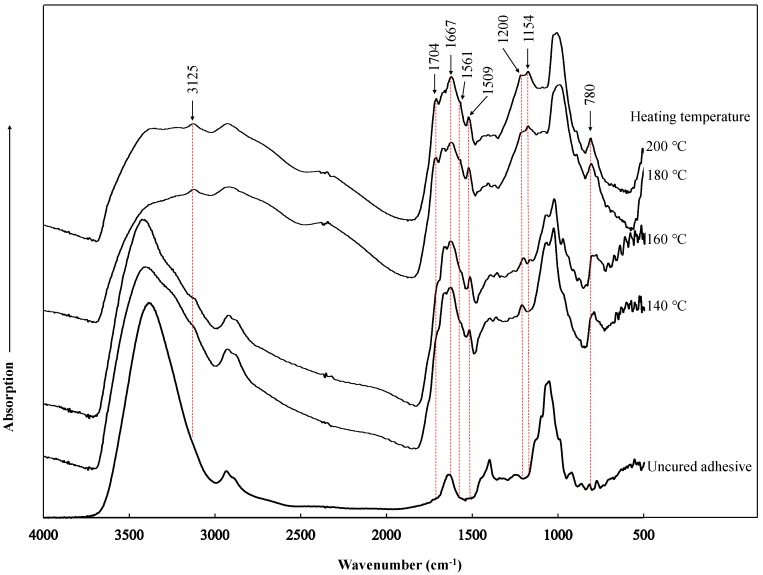
Infrared spectrum of the insoluble mass obtained from SADP 85/15 heated at different temperature and uncured adhesive obtained by drying at 80 °C for 15 h.

Figure 8 shows the adsorption bands exhibited by the insoluble masses obtained from the SADP 85/15 adhesives which had been heated at different temperatures. Compared to the spectra of the uncured adhesive, 6 new peaks and 1 shoulder were observable, and 1 peak clearly increased in the cured adhesives. Based on our previous research, the new peaks located at around 3125, 1509 cm^−1^ and the increased peak located at 780 cm^−1^ were possible due to the C–H, C=C stretching vibration and the CH=CH of furan ring, respectively [23,32,33], considering the 5-HMF itself is a soluble compound, the existence of furan ring in the insoluble mass indicated 5-HMF participated the curing reaction. The peaks located at 1704 and 1667 cm^−1^ were attributed to C=O derived from carbonyl functionalities [34], which were possible due to the dehydration of sucrose. Another peak observable at 1200 cm^−1^ was possible attributed to dimethylene ether bridges (–CH2OCH2–) [35]. These peaks indicated that the cured SADP 85/15 adhesive contains furan ring, carbonyl group and dimethylene ether bridges, which were possibly formed by the dehydration of sucrose, while ADP seems to act as a catalyst in the adhesion system. In addition, a new peak located at 1154 cm^−1^ and a shoulder located at around 1561 cm^−1^ were also observed through the addition of ADP. The shoulder at 1561 cm^−1^ was possibly attributed to the N–H stretching and bending vibration [36,37], and the peak at around 1154 cm^−1^ was possibly attributed to C–N bonds [38]. It seems that nitrogen participated in the polycondensation reaction of SADP adhesive, and this was possible due to the creation of ammonia from the decomposition of ADP. However, it is hard to recognize the existence of nitrogen in the cured adhesive by FT-IR analysis only, further research is needed to clear the chemical structure of the cured SADP adhesive.

### 3.6. Adhesive Curing Mechanism Considerations

The chemical reactions involved in thermal degradation of sucrose are a very complex network of potential reactions, therefore it is very difficult to elucidate all of the chemical structures formed in the cured SADP 85/15 adhesive. An overview of possible reactions taking place during the curing process could be described as follows. A primary reaction involving sucrose pyrolysis to glucose and fructose, in which the ADP may be acting as a catalyst [39]. Secondly, fructose is dehydrated and converted to 5-HMF. Meanwhile (and possibly concurrently), ADP decomposes to yield phosphoric acid and ammonia. Thirdly, the ammonia reacts with glucose and 5-HMF. In this process, Amadori rearrangement occurs which leads to a furanic product with an amine methyl at C2 and a hydroxymethyl at C5 [40]. It is also possible that 5-HMF converts to a Schiff base. Finally, an intense network of polycondensation reactions take place between the products of Amadori rearrangement and the Schiff base, which occurred under the system’s acidic environment. 

## 4. Conclusions

A novel eco-friendly wood adhesive which contains sucrose and ammonium dihydrogen phosphate (ADP) was developed for incorporation into particleboard products. Effects of mixture proportion and hot pressing temperature on the properties of the particleboards were investigated. When particleboard was bonded using SADP adhesive (85/15 proportion) with 200 °C hot pressing temperature, the properties of the final particleboard basically fulfilled the requirement of JIS A 5908 18 type standard, however this occurred at production conditions in this step of development as reported here, which were still outside usual industrial conditions. In addition, the general shape and weight of the particleboards were maintained throughout the stages of the cyclic aging treatments, indicating the boards possessed excellent water resistance. Results from FT-IR elucidate that a complex of polycondensation reactions took place during board curing, involving furanic ring, carbonyl groups and dimethylene ether bridges.

In this research, the resin content and hot pressing time are set at a relatively high level. However, considering this is a novel adhesion system, the experiments such as the effects of hot pressing time, resin content, chemical analysis, and improvement methods would be presented in our further research.

## Figures and Tables

**Table 1:** Viscosity and pH of SADP adhesive solutions.

Proportion (Sucrose/ADP)	Concentration (wt %)	Viscosity (mPa·s)	pH
100/0	50	22.5	6.8
95/5		22.3	3.9
90/10		21.7	3.8
85/15		20.3	3.8
80/20		19.6	3.7

**Table 2:** Manufacture conditions of the particleboards.

Group	SADP (Sucrose/ADP)	Hot Pressing Temperature (°C)	Hot Pressing Time (min)	Resin Content (wt %)
	100/0			
	95/5			
(a)	90/10	180	10	20
	85/15			
	80/20			
		140		
		160		
(b)	85/15	180	10	20
		200		
		220		

**Table 3:** pH of the solution after water-immersion treatment at 20 °C for 24 h.

Proportion (Sucrose/ADP)	pH of Soaked Water
100/0	4.9
95/5	3.7
90/10	3.1
85/15	2.8
80/20	2.6
